# Public Perceptions of Community Pharmacy-Based Naloxone Services: A National Cross-Sectional Survey

**DOI:** 10.3390/pharmacy10060171

**Published:** 2022-12-09

**Authors:** Lindsey A. Hohmann, Zach Krauss, Jitisha Patel, Grace T. Marley

**Affiliations:** 1 Harrison College of Pharmacy, Auburn University, 2316 Walker Building, Auburn, AL 36849, USA; 2 Health Sciences Center, School of Pharmacy, Cedarville University, 251 N Main St., Cedarville, OH 45314, USA; 3 Bernard J Dunn School of Pharmacy, Shenandoah University, 1775 N Sector Ct, Winchester, VA 22601, USA; 4 Eshelman School of Pharmacy, University of North Carolina (UNC), 301 Pharmacy Ln, Chapel Hill, NC 27599, USA

**Keywords:** community pharmacy, naloxone, opioids, substance abuse, perceptions, cross-sectional survey, communication, health outcomes research

## Abstract

Little is known about the general public’s perceptions regarding community pharmacist-delivered naloxone services at the national level. Accordingly, the purpose of this study was to describe the US general public’s awareness, knowledge, beliefs, comfort, perceived barriers, abilities, and communication preferences related to community pharmacy-based naloxone services. A national, online cross-sectional survey was conducted in September 2021 among US adults ≥18 years recruited via Amazon Mechanical Turk (MTurk). Primary outcome measures were assessed via 5-point Likert-type scales, including: (1) naloxone awareness and knowledge; (2) naloxone beliefs; (3) comfort with pharmacist-provided naloxone; (4) perceived barriers to pharmacy-based naloxone; (5) opioid overdose competencies, concerns, and readiness; and (6) preferred pharmacist-patient naloxone communication strategy. Analyses included descriptive statistics and logistic regression models to assess predictors of preferred communication strategies. Of 301 respondents, 82.1% were White, 48.8% female, and mean 43 years. Eighty-five percent were unaware of pharmacy-provided naloxone and mean [SD] knowledge score was low (29.3% [16.8]). Mean [SD] beliefs (3.78 [0.61]) and comfort (3.70 [0.54]) were positive, while perceived barriers were low/neutral (2.93 [0.78]). For communication, 54% preferred general advertisement, 32.9% universal offer, and 13.3% targeted offer. The odds of preferring a general advertisement or universal offer over a targeted offer increased with greater awareness (AOR:4.52; *p* = 0.003) and comfort (AOR:3.79; *p* = 0.003), and decreased with greater competence (AOR:0.35; *p* = 0.001). Although awareness and knowledge regarding community pharmacy-based naloxone services was low, beliefs and comfort were positive and perceived barriers were low/neutral. General or universal offers of naloxone were preferred over targeted approaches. Future studies should test the impact of communication strategies on naloxone uptake.

## 1. Introduction

Opioid misuse is a major public health issue in the US. According to provisional data from the Center for Disease Control and Prevention, an estimated 80,816 drug overdose deaths occurred due to opioids in 2021 [1]. This encompasses overdoses due to illicit opioids such as heroin, as well as prescription opioid analgesics [2]. Naloxone is an opioid antagonist that is designed to reverse an opioid overdose [3], and is 75–100% effective in reviving an individual experiencing an overdose when administered by a bystander [4]. Therefore, efforts to increase access to, public awareness, and public acceptance of naloxone remain critical in order to mitigate opioid overdose fatalities.

Community pharmacists offer a solution to the issue of opioid misuse and overdose through readily available, no-cost education on naloxone in its various available forms [5]. Community pharmacists in each US state are able to dispense naloxone without a physician visit according to state-specific policies, including statewide standing orders or pharmacists’ prescriptive authority [6]. In general, US community pharmacists are willing to participate in naloxone dispensing and education [5]. Furthermore, studies show that pharmacist-provided education and distribution can improve access, utilization, and harm reduction in the communities where naloxone is dispensed [7]. However, despite the success of some pharmacy-based naloxone interventions [8,9], pharmacy-based naloxone services have not reached capacity in the US [10] and perceived patient resistance to naloxone dispensing remains a persistent barrier reported by pharmacists [10,11].

In order to develop effective strategies to overcome perceived patient resistance to pharmacy-based naloxone services, it is necessary to better understand public perceptions and preferences that may be contributing to this resistance. However, little is known about the general public’s perceptions regarding community pharmacist-delivered naloxone services at the national level [12], with most available literature focusing on niche patient populations who have experienced overdose in the past or those being discharged from the emergency department [13]. Specifically, to date, there is a sparsity in the literature related to patient perceptions or preferences regarding naloxone communication strategies within the community pharmacy [14,15]. State-level studies have shown mixed reactions towards naloxone services and the presence of knowledge gaps about naloxone among the public. For example, a survey distributed in Virginia showed that most of the general public favored pharmacy-delivered naloxone services but were not initially aware of naloxone and had concerns of naloxone promoting drug misuse [16]. Another survey distributed in Nebraska showed respondents having little knowledge of naloxone and having stigmatizing beliefs about unsafe drug use with naloxone [17]. While these studies show mixed perceptions and limited knowledge about naloxone, understanding the general public’s perceptions at the national level could provide greater insight into knowledge gaps that can be leveraged in future studies and educational interventions in order to enhance pharmacy-based naloxone services. Therefore, the purpose of this study was to describe the US general public’s awareness, knowledge, beliefs, comfort, perceived barriers, abilities, and communication preferences related to community pharmacy-based opioid counseling and naloxone services.

## 2. Materials and Methods

### 2.1. Study Design and Participant Recruitment

A national cross-sectional survey study was conducted in September 2021. Inclusion criteria consisted of: (1) age ≥ 18 years; (2) living in the United States; and (3) able to understand the English language. Participants were recruited using an Amazon Mechanical Turk (MTurk) online panel. MTurk is an online platform used for survey distribution and recruitment with the ability to reach an audience of more than 500,000 people [18]. The majority of the MTurk participant pool is located in the United States and has been shown to closely reflect the demographics of the US population in terms of age, race, and sex [19,20]. In order to ensure robust survey findings, participants were restricted to US residents who had achieved MTurk “Master” status, indicating a history of high-quality responses [20,21]. Individuals received financial compensation of $5 upon survey completion.

### 2.2. Sample Size Calculation

An a priori power calculation was conducted using G*Power version 3.1.9.7 (Heinrich-Heine-Universität Düsseldorf, Düsseldorf, Germany) [22]. Assuming an alpha of 0.05 and effect size of 1.85 (expected odds ratio) [23], a sample size of 132 was expected to be sufficient to assess primary outcome measures (predictors of naloxone communication preference) with 80% power. The final survey sample size was adequate to meet this need (see Section 3.1).

### 2.3. Data Collection and Measures

An anonymous electronic survey was distributed to eligible US participants via MTurk. The 74-item survey instrument was developed de novo and informed by Smith’s (2019) [12] naloxone belief scale, Nielsen’s (2016) [24] comfort and barrier scales, and Williams’ (2013) [25] opioid overdose knowledge scale (OOKS) and attitude scale (OOAS). Outcome measures included: (1) general awareness and knowledge about opioid overdose and naloxone; (2) general beliefs about naloxone; (3) comfort with community pharmacist-led naloxone provision; (4) perceived barriers to pharmacy-based naloxone access; (5) competencies, concerns, and readiness regarding managing an opioid overdose; and 6) preferred pharmacist-patient naloxone communication strategy. General awareness (2-items) and knowledge (8-items) were assessed via closed-ended dichotomous (Yes/No) and multiple-choice indices, respectively. General beliefs about naloxone (6-items), comfort (11-items), barriers (7-items), competence (10-items), concerns (8-items), and readiness (10-items) were measured using 5-point Likert-type scales from 1 = strongly disagree to 5 = strongly agree. Preferred pharmacist-patient naloxone communication strategy was measured using a 1-item closed-ended multiple choice question with three response categories, including general advertisement (flyer or brochure posted in the pharmacy), universal offer (recommendation from the pharmacist to all patients receiving an opioid prescription, regardless of perceived overdose risk), or targeted offer (recommendation from the pharmacist to patients at higher risk of opioid overdose only).

History of prescription opioid use (1-item; e.g., “*Are you currently using prescription opioid medications or have you been prescribed opioid medications in the past for the treatment of pain or for post-surgery management?*”) and life exposure to opioid use disorder (1-item; e.g., “*Have you or someone you have known been personally or professionally affected by opioid use disorder (OUD) or substance misuse?*”) were also collected via closed-ended dichotomous (yes/no) multiple choice questions. The survey instrument was pre-tested for face and content validity amidst members of the primary author’s department (*n* = 2), and question wording and flow was revised based on feedback prior to distribution via MTurk. See Supplementary File S1 for a copy of the full survey instrument.

### 2.4. Data Analysis

Demographics and measures were characterized using descriptive statistics. Mean knowledge scores were calculated based on the percent of items in the knowledge index for which participants provided a correct response. General beliefs, comfort, barriers, competence, concerns, and readiness scale items were summed and averaged to create overall scale scores. Scale items were reverse coded as necessary such that higher scale means indicated more of the construct (e.g., greater comfort, more barriers, more concerns, etc), and internal consistency of constructs was measured using KR-20 (knowledge) or Cronbach’s α. Differences in ability to manage an opioid overdose (mean competence, concerns, and readiness scale scores) were compared between those with and without a history of prescription opioid use and life exposure to opioid misuse via two-sided Mann–Whitney U tests with an a priori alpha level of 0.05.

Predictors of preferred patient-pharmacist naloxone communication strategy were also assessed using logistic regression models. Naloxone communication strategy (dependent variable) was dichotomized into “General Advertisement or Universal Offer” and “Targeted Offer.” Predictor variables included knowledge, general naloxone beliefs, comfort, barriers, competence, concerns, and readiness mean scale scores, as well as awareness of naloxone in general and pharmacist-provided naloxone specifically (Yes/No). In order to determine if model outcomes were influenced by sociodemographic factors, two models were assessed: (1) unadjusted (Model 1), excluding sociodemographic covariates; and (2) adjusted (Model 2), controlling for age, gender, ethnicity, race, education, annual household income, history of opioid use, and life exposure to opioid misuse. All analyses were performed with SPSS Statistics software version 27 (IBM Corp, Armonk, NY, USA). Study procedures were approved via the Institutional Review Board (IRB) at the primary author’s institution (Protocol No. 21-336 EX 2107) and all survey respondents consented to participate.

## 3. Results

### 3.1. Respondent Characteristics

Overall, 301 surveys were completed, with an average completion time of 26 min. The majority of respondents were White (82.1%), male (50.5%), with a mean age of 43 years (Table 1). Participants resided throughout 44 US states, with the largest percentage (9.6%) located in California. Over 35% had a history of prescription opioid use, and 40% had life exposure to opioid use disorder.

### 3.2. Awareness and Knowledge about Opioid Overdose and Naloxone

Public awareness of naloxone was low, as about 22% of participants had never heard of naloxone, and 85% were unaware that naloxone can be obtained from a community pharmacy without a physician visit (Table 2). Furthermore, overall knowledge (KR-20 = 0.515) about naloxone and management of opioid overdose was low, with only mean (SD) 29.3% (16.8) of questions answered correctly. Naloxone’s onset and duration of action were correctly identified by 44.2% and 15% of respondents, respectively. While 37.2% correctly identified opioid overdose risk factors, only 13.6% correctly reported the actions to take when managing an opioid overdose and 94.4% did not know how naloxone was administered.

### 3.3. General Beliefs about Naloxone

Beliefs about naloxone (Cronbach’s α = 0.500) tended to be positive with a mean (SD) scale score of 3.78 (0.61) (Table 3). Specifically, 53.5% strongly agreed that naloxone provides opioid users a second chance at life and 58.5% strongly agreed that naloxone should be available for anyone concerned about opioid overdose. While only 7.6% strongly agreed that naloxone enables people who misuse opioids, about 22% stated that they personally would not need naloxone if prescribed opioids.

### 3.4. Comfort with Community Pharmacist-Led Naloxone Provision

Comfort (α = 0.705) with pharmacist-delivered naloxone was generally positive with a mean (SD) scale score of 3.70 (0.54) (Table 4). For example, 49.2% strongly agreed that pharmacists have sufficient knowledge to dispense naloxone and 63.8% supported community pharmacists dispensing naloxone. On the other hand, 19.3% stated that they would only get naloxone from the pharmacy if their doctor recommended it first.

### 3.5. Perceived Barriers to Utilizing Community Pharmacy-Based Naloxone Provision

Perceived barriers (α = 0.753) to utilizing pharmacist-delivered naloxone were low/neutral with a mean (SD) scale score of 2.93 (0.78) (Table 5). The most frequently reported barrier was discomfort asking the pharmacist for naloxone (65.5% agreed or strongly agreed), followed by not wanting their neighborhood/community (54.1%) or family/friends (53.2%) to see them getting naloxone. The least frequent barrier was long wait-times to receive naloxone from the pharmacy (14.3%).

### 3.6. Ability to Manage an Opioid Overdose

#### 3.6.1. Self-Reported Competence, Concerns, and Readiness Regarding Opioid Overdose Management

Overall, self-reported competence (α = 0.901) in managing an opioid overdose was low with a mean (SD) scale score of 2.61 (0.85) (Table 6a). For example, only 15.6% of respondents agreed or strongly agreed that they would be able to effectively handle an opioid overdose and 13.6% stated that they felt able to administer naloxone to an overdosed individual.

However, concerns (α = 0.708) regarding intervening during an overdose were fairly low/neutral (mean [SD]: 2.64 [0.71]) (Table 6b), and readiness (α = 0.869) to intervene was high (mean [SD]: 4.11 [0.63]) (Table 6c). In particular, major concerns included accidentally hurting someone who overdosed (50.9%) and doing something wrong during an overdose (76.7%). In regard to readiness, 92.0% reported that they want to be able to help someone who has overdosed and 74.4% stated that they would do whatever was necessary to save an overdose victim.

#### 3.6.2. Effect of History of and Exposure to Opioid Use/Misuse on Competence, Concerns, and Readiness Regarding Opioid Overdose Management

Among those with a history of past or current prescription opioid use, mean (SD) competence in managing an opioid overdose was higher compared to those without a history of opioid use (2.80 [0.89] vs. 2.49 [0.80], *p* = 0.003) (Table 7a). Additionally, concerns regarding opioid overdose management were lower in those with versus without a history of opioid use (2.54 [0.70] vs. 2.70 [0.70], *p* = 0.047). However, there was no statistically significant difference in readiness regarding opioid overdose management.

Furthermore, prior life exposure to opioid or substance misuse led to statistically significant differences in competence, concerns, and readiness to manage an opioid overdose (Table 7b). Specifically, those with prior exposure had higher mean (SD) competence (2.91 [0.82] vs. 2.40 [0.80], *p* < 0.001), lower concerns (2.66 [0.65] vs. 2.89 [0.65], *p* = 0.001), and higher readiness (4.28 [0.58] vs. 4.01 [0.63], *p* < 0.001) when compared to their counterparts with no prior exposure to opioid misuse.

### 3.7. Patient-Pharmacist Naloxone Communication Preferences

#### 3.7.1. Proportion of Respondents Preferring General, Universal, or Targeted Naloxone Communication Strategies

In terms of patient-pharmacist naloxone communication strategies, the majority of participants (53.8%) preferred a general advertisement (flyer or poster in the pharmacy) and 32.9% preferred a universal offer of naloxone (recommendation from the pharmacist to any patients receiving prescription opioids). Only 13.3% preferred a targeted offer from the pharmacist exclusively to patients at higher risk of opioid overdose.

#### 3.7.2. Factors Predicting Patient-Pharmacist Naloxone Communication Strategy Preference

Three factors predicted patient-pharmacist naloxone communication style preference: awareness of naloxone in general; comfort with pharmacist-provided naloxone services; and competence in managing an opioid overdose (Table 8). Specifically, the odds of preferring a general advertisement or universal offer over a targeted offer of naloxone increased with greater awareness of naloxone in general (AOR: 4.52, 95% CI: 1.65–12.40; *p* = 0.003) and comfort with pharmacist-provided naloxone (AOR: 3.79, 95% CI: 1.58–9.07; *p* = 0.003), and decreased with greater competence in managing an opioid overdose (AOR: 0.35, 95% CI: 0.19–0.65; *p* = 0.001). Model outcomes did not differ between the unadjusted (Model 1; R^2^: 0.16) and adjusted models (Model 2; R^2^: 0.25).

## 4. Discussion

Overall, the mix of respondent demographics in the current study was similar to the demographics of the US population as a whole [19]. This study investigated public awareness, knowledge, beliefs, comfort, barriers, abilities, and communication preferences regarding naloxone at the national level. To date, the majority of research in this vein has been at the state level [14,16,17], with little focused on a national scale [12]. Furthermore, unique to this study is a description of the influential factors affecting individuals’ preference for community pharmacy-based naloxone communication strategies.

Awareness and knowledge regarding naloxone were relatively low amongst the US general public. Although 88% had previously heard of naloxone, there remains a gap in awareness amongst 22% of respondents, which may represent a clinically significant proportion of individuals who are not being reached by current naloxone public health campaigns and interventions. This is consistent with previous research that found 61% of US adults had heard of naloxone in 2017 [12]. Despite an apparent increase in naloxone awareness between 2017 and the current study in 2021, a gap in awareness remains [12]. Furthermore, although beliefs regarding naloxone were generally positive, knowledge on how to take action in the case of an opioid overdose was low. This aligns with previous research that found positive beliefs towards naloxone at the national level [12] but limited knowledge on how to use it [17]. However, beliefs in rural US regions have been fairly negative [16], indicating potential gaps in knowledge dissemination. In light of this, additional efforts to fill these awareness and knowledge gaps, such as partnerships between academicians/researchers and community-based organizations like recreation centers and adult extension learning facilities that have access to hard-to-reach populations that do not seek healthcare regularly, are warranted [26]. Specifically, future efforts should focus on providing public education surrounding the steps to take when managing an opioid overdose and how to administer naloxone.

In regard to community pharmacy-delivered naloxone, public comfort was fairly high in the current study (rated 3.70 out of 5 on average). Similar to previous studies [16], while most individuals in the current study were supportive of pharmacist-delivered naloxone and comfortable receiving naloxone in a pharmacy setting, concerns remained regarding community pharmacists’ level of knowledge about naloxone. A fairly large percentage of participants (almost 20%) also stated that they would only get naloxone from the pharmacy if their physician recommended it first. This suggests that additional education is warranted in order to clarify pharmacists’ credentials and role in the healthcare team; partnerships between practitioners and local schools and colleges of pharmacy can assist with developing and disseminating public education campaigns.

In addition, perceived barriers to utilization of community pharmacy-based naloxone services were centered around social factors in the current study. For example, major barriers revolved around discomfort asking the pharmacist about naloxone and fear of neighbors, family, or friends finding out. This fear of perceived stigma has been reported in multiple previous studies regarding naloxone provision [7,14,27,28,29], and points toward the need for organizational changes within community pharmacy operations such as storewide loudspeaker advertisements, posters, and bag stuffers in order to reduce stigma and normalize provision of naloxone [10,30].

Overall, respondents felt that they were ready to participate in opioid overdose management with naloxone, but many expressed concerns related to fear, reluctance, inadequate training, and inability to carry out the intervention well. These concerns were less common in those with a history of opioid use or those who had a history of life exposure to opioid misuse. Similar findings have been reported amongst friends and family members of people who use heroin [31]. Future studies should investigate the impact of incorporating testimonials from caregivers and people who use opioids into naloxone education programs.

In terms of communication preferences, respondents preferred a general advertisement (such as a flyer or brochure) or universal offer of naloxone in comparison to a targeted offer from their pharmacist. Respondents were more likely to prefer a general advertisement or universal offer of naloxone in the pharmacy if they had higher awareness of naloxone or greater comfort with pharmacist-provided naloxone. It is interesting to note that although the largest percentage of individuals preferred a general advertisement strategy, this strategy necessitates an individual to then approach their pharmacist and ask for naloxone, and this study also found that 65.5% of respondents agreed or strongly agreed that it is uncomfortable asking a pharmacist for naloxone. As mentioned previously, strategies such storewide loudspeaker advertisements, posters, and bag stuffers to normalize provision of naloxone [10,30] may increase patient comfort with approaching their pharmacist about naloxone and aid in overcoming this barrier. An alternative, universal offer to all patients who filled opioid prescriptions was preferred by 32.89% of respondents. This is similar to findings from focus groups by Green et al. that found respondents preferred a universal opt-out strategy in which all patients meeting certain criteria are offered naloxone versus a targeted or opt-in strategy that requires patients to request naloxone [14]. This universal offer would take some of the social barriers out of a request for naloxone, as 39.6% of respondents agreed that they feared the pharmacist having a negative view of them after requesting naloxone. Through creating a universal opt-out approach to naloxone provision in community pharmacies, we can ease the logistical burden associated with targeted models of distribution.

This study has the potential to impact public health on a broader scale by providing insight that can inform future interventions in clinical practice at the organization, state, and national levels. At the organizational level, researchers and academician can help to connect clinicians with community-based organizations that serve traditionally hard-to-reach patients, providing an opportunity to narrow the knowledge gap by providing naloxone training and education at venues such as recreation centers and adult extension learning facilities [26]. Furthermore, community pharmacy organizations can normalize naloxone provision by increasing their customer-facing naloxone promotional materials, including announcements, posters, and flyers, as well as training for pharmacists on how to effectively communicate and recommend naloxone in a universal and non-stigmatizing manner [29]. At the state level, researchers, academicians, and clinicians can partner with departments of public health to develop and disseminate public awareness campaigns surrounding naloxone (e.g., freeway billboards, demonstration projects) [30]. Nationally, professional pharmacy organizations can partner with academicians, researchers, and clinicians to develop and distribute naloxone education materials and communication best practice guidelines that are tailored for the community pharmacy setting [10].

### Limitations

Several limitations of this study must be noted. First, the cross-sectional design prohibits drawing causal assumptions from this study’s findings. Future studies should assess the public’s naloxone knowledge and perceptions over time in order to identify and control for additional potential confounders such as policies and seasonal effects. On a related note, the current study could not control for all outside influences on respondents’ naloxone knowledge, awareness, and perceptions, and it is possible that unmeasured factors may offer further explanation regarding naloxone communication preferences. In light of the low variation in model outcomes explained by the predictors measured in this study (R^2^ = 0.25), follow-up qualitative studies are necessary in order to expand upon and probe further into the influential factors affecting choice of preferred naloxone communication strategy. Furthermore, as the population reached via MTurk was limited to those with Internet access, study findings may not be generalizable to those in rural or disadvantaged areas. However, the demographic characteristics of study participants were similar to that reported in other MTurk studies [19] and to the US as a whole. Lastly, selection bias is a concern, as those interested in the topic of opioid misuse may have self-selected to take the survey. Future studies may distribute the survey instrument used in the current study via multiple channels and platforms in order to compare findings to those gathered through MTurk; however, MTurk is a feasible distribution platform that enables a national audience to be reached [18,19,20].

## 5. Conclusions

Although awareness and knowledge regarding community pharmacy-based naloxone services was low, beliefs and comfort were positive among the US general public. Perceived barriers to utilization of community pharmacy-based naloxone services were low/neutral and tended to focus on social factors related to fear of perceived stigma surrounding naloxone. Similarly, general advertisements (flyer or brochure posted in the pharmacy) or universal offers (recommendation from the pharmacist to all patients receiving an opioid prescription, regardless of perceived overdose risk) of naloxone from the pharmacist were preferred over more targeted communication approaches in the US. Naloxone communication preferences were further influenced by level of naloxone awareness, comfort with pharmacist-provided naloxone, and perceived competence in in managing an opioid overdose. Findings from the current study may inform future studies testing the impact of various communication strategies on naloxone uptake.

## Figures and Tables

**Table 1:** Participant Demographics (*n* = 301).

Characteristics	*n* (%) ^a^
**Sex**	
Male	152 (50.5)
Female	147 (48.8)
**Race**	
White/Caucasian	247 (82.1)
Black/African American	27 (9)
Asian or Pacific Islander	20 (6.6)
**Ethnicity**	
Hispanic origin	14 (4.7)
Non-Hispanic origin	285 (94.7)
**Employment**	
Non-healthcare Professional	248 (82.4)
Healthcare Professional	9 (3)
Unemployed	44 (14.6)
**Education**	
High school graduate, diploma or equivalent	47 (15.6)
Trade/technical/vocational training	14 (4.7)
Some college credit, no degree	46 (15.3)
Associate degree	38 (12.6)
Bachelor’s degree	123 (40.9)
Master’s degree	26 (8.6)
Doctorate degree	2 (0.7)
Professional degree	5 (1.7)
**Annual Household Income**	
Less than $20,000	38 (12.6)
$20,000–$49,999	114 (37.9)
$50,000–$99,999	107 (35.5)
$100,000–$149,999	29 (9.6)
$150,000 or more	13 (4.3)
**Life Exposure to Opioid Use Disorder**	
“*Have you or someone you have known been personally or professionally affected by opioid use disorder (OUD) or substance misuse?*”	
Yes	120 (40)
No	180 (60)
**History of Prescription Opioid Use**	
“*Are you currently using prescription opioid medications or have you been prescribed opioid medications in the past for the treatment of pain or for post-surgery management?*”	
Yes	108 (35.9)
No	193 (64.1)
	**Mean (SD)**
**Age, years**	43.1 (10.7)

^a^ Percentages may differ due to item non-response.

**Table 2:** General Public’s Knowledge and Awareness Regarding Opioid Overdose and Naloxone (*n* = 301).

Knowledge and Awareness Indices	Mean (SD)
**Overall Knowledge Score (% Questions Answered Correctly)**	29.34 (16.76)
**Knowledge Items**	***n* (%) ^a^**
What is naloxone used for? *Correct response(s): To reverse the effects of an opioid overdose (e.g., heroin, Oxycodone, Vicodin, methadone)*	286 (95.0)
Which of the following factors increase the risk of an opioid overdose? ^b^ *Correct response(s): Taking larger than usual doses of opioids; Increase in opioid purity; Using an opioid again after not having used for a while; Using an opioid when no one else is around; A long history of opioid use; Using an opioid again after a detoxification treatment*	112 (37.2)
Which of the following are indicators of an opioid overdose? ^b^ *Correct response(s): Having bloodshot eyes; Slow or shallow breathing; Lips, hands or feet turning blue; Loss of consciousness; Unresponsive; Deep snoring; Very small pupils; Agitated behavior; Rapid heartbeat*	18 (6)
Which of the following should be done when managing an opioid overdose? ^b^ *Correct response(s): Call an ambulance; Stay with the person until an ambulance arrives; Give mouth to mouth resuscitation; Place the person in the recovery position (on their side with their mouth clear); Give naloxone (opioid overdose antidote); Check for breathing; Check for blocked airways (nose and mouth)*	41 (13.6)
How can naloxone be administered? ^b^ *Correct response(s): Into the nose (intranasal); Injected into a muscle (intramuscular); Injected into a vein (intravenous); Injected under the skin (subcutaneous)*	17 (5.6)
How long does naloxone take to start having an effect?	
*Correct response(s): 2–5 min*	133 (44.2)
How long do the effects of naloxone last for?	
*Correct response(s): About 1 h*	45 (15)
Which statement(s) are CORRECT about naloxone and opioid overdose? ^b^ *Correct response(s): If the first dose of naloxone has no effect then a second dose can be given; Someone can overdose again even after having received naloxone; Naloxone can provoke withdrawal symptoms*	38 (10)
**Awareness Items**	***n* (%) ^a^**
Before taking this survey, were you aware of naloxone, also commonly referred to as Narcan^®^, and what it is used for?	
Yes	235 (78.1)
No	66 (21.9)
Naloxone, or Narcan^®^ is a medication used for the treatment of opioid overdose and can help to reverse an opioid overdose. Were you aware that in all 50 states you can obtain this medication without a prescription by speaking with your pharmacist?	
Yes	45 (15)
No	256 (85)

^a^ Frequencies and percentages may differ due to item non-response. ^b^ Respondents were asked to select all answers that applied.

**Table 3:** General Beliefs About Naloxone (*n* = 301).

Belief Items	*n* (%)				
	1	2	3	4	5
Naloxone provides opioid users with a second chance at life	1 (0.3)	4 (1.3)	35 (11.6)	100 (33.2)	161 (53.5)
Naloxone may cause more overdoses ^a^	105 (34.9)	87 (28.9)	80 (26.6)	20 (6.6)	9 (3.0)
Having Naloxone available enables people who misuse opioids ^a^	97 (32.2)	79 (26.2)	63 (20.9)	39 (13.0)	23 (7.6)
Naloxone should be made available for anyone concerned about opioid overdose	3 (1.0)	8 (2.7)	45 (15.0)	69 (22.9)	176 (58.5)
If my pharmacist speaks to me about Naloxone, I would like to be moved to a private area in the pharmacy	33 (11.0)	35 (11.6)	62 (20.6)	62 (20.6)	109 (36.2)
If my doctor prescribed me opioids, I would not need Naloxone ^a^	48 (15.9)	49 (16.3)	90 (29.9)	48 (15.9)	66 (21.9)
	**Mean (SD)**				
**Overall Belief Scale Score**	3.78 (0.61)				

1 = Strongly disagree, 2 = Disagree, 3 = Neutral, 4 = Agree, 5 = Strongly agree. ^a^ Item reverse-coded.

**Table 4:** Participant Comfort with Pharmacist Provision of Naloxone (*n* = 301).

Comfort Items	*n* (%)				
	1	2	3	4	5
I am confident that pharmacists have sufficient KNOWLEDGE to dispense naloxone	2 (0.7)	9 (3.0)	30 (10.0)	112 (37.2)	148 (49.2)
I am confident that pharmacists have sufficient TRAINING to dispense naloxone	3 (1.0)	18 (6.0)	28 (9.3)	106 (35.2)	146 (48.5)
I support community pharmacists dispensing naloxone	5 (1.7)	6 (2.0)	38 (12.6)	60 (19.9)	192 (63.8)
I prefer receiving naloxone from my pharmacist rather than my doctor	17 (5.6)	28 (9.3)	163 (54.2)	52 (17.3)	41 (13.6)
I would only get naloxone from my pharmacy if my doctor recommended it first ^a^	37 (12.3)	66 (21.9)	74 (24.6)	66 (21.9)	58 (19.3)
If I received naloxone from my pharmacy, I would NOT want them to tell my doctor ^a^	117 (38.9)	80 (26.6)	62 (20.6)	21 (7.0)	21 (7.0)
I prefer to receive naloxone from a more clinical setting, like a doctor’s office ^a^	31 (10.3)	76 (25.2)	126 (41.9)	42 (14.0)	26 (8.6)
The pharmacy is an appropriate place to provide naloxone	3 (1.0)	8 (2.7)	38 (12.6)	87 (28.9)	165 (54.8)
I worry how the pharmacy will handle my naloxone records ^a^	86 (28.6)	87 (28.9)	55 (18.3)	48 (15.9)	25 (8.3)
There is not enough privacy in the pharmacy ^a^	53 (17.6)	81 (26.9)	95 (31.6)	49 (16.3)	23 (7.6)
I would prefer to receive naloxone from an anonymous source, like the public health department ^a^	67 (22.3)	67 (22.3)	112 (37.2)	39 (13.0)	16 (5.3)
	**Mean (SD)**				
**Overall Comfort Scale Score**	3.70 (0.54)				

1 = Strongly disagree, 2 = Disagree, 3 = Neutral, 4 = Agree, 5 = Strongly agree. ^a^ Item reverse-coded.

**Table 5:** General Public’s Perceived Barriers to Utilizing Pharmacist-Delivered Opioid Counseling and Naloxone Services (*n* = 301).

Barriers Items	*n* (%)				
	1	2	3	4	5
Cost of Naloxone is too high	28 (9.3)	39 (13)	124 (41.2)	77 (25.6)	33 (11)
It is uncomfortable asking the pharmacist for naloxone	27 (9)	35 (11.6)	42 (14)	132 (43.9)	65 (21.6)
Transportation to the pharmacy is difficult	135 (44.9)	77 (25.6)	41 (13.6)	39 (13)	9 (3)
Not wanting my family/friends to know that I was getting naloxone	36 (12)	59 (19.6)	46 (15.3)	105 (34.9)	55 (18.3)
Not wanting people I know from my community/neighborhood to see me getting naloxone	37 (12.3)	56 (18.6)	45 (15)	97 (32.2)	6 (21.9)
Fear of the pharmacist having a negative view of me if I ask for naloxone	70 (23.2)	62 (20.6)	50 (16.6)	83 (27.6)	36 (12)
It will take too long to receive naloxone from the pharmacy (i.e., long wait times)	89 (29.6)	102 (33.9)	67 (22.3)	31 (10.3)	12 (4)
		**Mean (SD)**			
**Overall Barriers Scale Score**		2.93 (0.78)			

1 = strongly disagree; 2 = disagree; 3 = neutral; 4 = agree; 5 = strongly agree.

**Table 6:** (**a–c**) Ability to Manage an Opioid Overdose (*n* = 301): (**a**) Competence; (**b**) Concerns; and (**c**) Readiness.

**(a)**					
**Competence Items**	***n* (%)**				
	**1**	**2**	**3**	**4**	**5**
I already have enough information about how to manage an overdose	94 (31.2)	131 (43.5)	43 (43.5)	27 (9.0)	6 (2.0)
I am already able to inject naloxone into someone who has overdosed	148 (49.2)	79 (26.2)	33 (11.0)	29 (9.6)	12 (4.0)
I would be able to check that someone who has overdosed was breathing properly	26 (8.6)	43 (14.3)	62 (20.6)	121 (40.2)	49 (16.3)
I’m going to need more training before I would feel confident to help someone who has overdosed ^a^	8 (2.7)	20 (6.6)	24 (8.0)	83 (27.6)	166 (55.1)
I would be able to perform mouth to mouth resuscitation to someone who has overdosed	54 (17.9)	73 (24.3)	63 (20.9)	79 (26.2)	32 (10.6)
I would be able to perform chest compressions to someone who has overdosed	41 (13.6)	66 (21.9)	59 (19.6)	94 (31.2)	41 (13.6)
If someone overdoses, I would know what to do to help them	55 (18.3)	101 (33.6)	73 (24.3)	60 (19.9)	12 (4.0)
I would be able to place someone who has overdosed in the recovery position	31 (10.3)	37 (12.3)	55 (18.3)	99 (32.9)	79 (26.2)
I know very little about how to help someone who has overdosed ^a^	14 (4.7)	55 (18.3)	58 (19.3)	86 (28.6)	88 (29.2)
I would be able to deal effectively with an overdose	62 (20.6)	103 (34.2)	89 (29.6)	37 (12.3)	10 (3.3)
		**Mean (SD)**			
**Overall Competence Scale Score**		2.06 (0.85)			
**(b)**					
**Concern Items**	***n* (%)**				
	**1**	**2**	**3**	**4**	**5**
I would be afraid of giving naloxone in case the person becomes aggressive afterwards	64 (21.3)	108 (35.9)	52 (17.3)	65 (21.6)	12 (4.0)
I would be afraid of doing something wrong in an overdose situation	16 (5.3)	20 (6.6)	34 (11.3)	115 (38.2)	116 (38.5)
I would be reluctant to use naloxone for fear of precipitating withdrawal symptoms	78 (25.9)	121 (40.2)	57 (18.9)	34 (11.3)	11 (3.7)
I would be concerned about calling emergency services in case the police come around	149 (49.5)	73 (24.3)	35 (11.6)	32 (10.6)	12 (4.0)
If I tried to help someone who has overdosed, I might accidentally hurt them	27 (9.0)	44 (14.6)	77 (25.6)	111 (36.9)	42 (14.0)
I would feel safer if I knew that naloxone was around ^a^	8 (2.7)	22 (7.3)	107 (35.5)	99 (32.9)	65 (21.6)
I would be afraid of suffering a needle stick injury if I had to give someone a naloxone injection	73 (24.3)	96 (31.9)	60 (19.9)	42 (14.0)	30 (10.0)
Needles frighten me and I wouldn’t be able to give someone an injection of naloxone	110 (36.5)	87 (28.9)	56 (18.6)	29 (9.6)	19 (6.3)
		**Mean (SD)**			
**Overall Concerns Scale Score**		2.64 (0.71)			
**(c)**					
**Readiness Items**	***n* (%)**				
	**1**	**2**	**3**	**4**	**5**
Everyone at risk of witnessing an overdose should be given a naloxone supply	17 (5.6)	44 (14.6)	75 (24.9)	104 (34.6)	61 (20.3)
I couldn’t just watch someone overdose, I would have to do something to help	7 (2.3)	9 (3.0)	37 (12.3)	120 (39.9)	128 (42.5)
If someone overdoses, I would call an ambulance but I wouldn’t be willing to do anything else ^a^	74 (24.6)	110 (36.5)	65 (21.6)	33 (11.0)	19 (6.3)
Family and friends of drug users should be prepared to deal with an overdose	3 (1.0)	11 (3.7)	32 (10.6)	105 (34.9)	150 (49.8)
If I saw an overdose, I would panic and not be able to help ^a^	77 (25.6)	126 (41.9)	59 (19.6)	31 (10.3)	8 (2.7)
If I witnessed an overdose, I would call an ambulance straight away	-	10 (3.3)	20 (6.6)	69 (22.9)	202 (67.1)
I would stay with the overdose victim until help arrives	4 (1.3)	4 (1.3)	13 (4.3)	85 (28.2)	195 (64.8)
If I saw an overdose, I would feel nervous, but I would still take the necessary actions	2 (0.7)	9 (3.0)	40 (13.3)	119 (39.5)	131 (43.5)
I will do whatever is necessary to save someone’s life in an overdose situation	4 (1.3)	14 (4.7)	59 (19.6)	113 (37.5)	111 (36.9)
If someone overdoses, I want to be able to help them	2 (0.7)	5 (1.7)	17 (5.6)	108 (35.9)	169 (56.1)
		**Mean (SD)**			
**Overall Readiness Scale Score**		4.11 (0.63)			

1 = Strongly disagree, 2 = Disagree, 3 = Neutral, 4 = Agree, 5 = Strongly agree. ^a^ Item reverse-coded.

**Table 7:** (**a**) Effect of history of opioid use on ability to manage an opioid overdose (*n* = 301); and (**b**) Effect of prior life exposure to opioid misuse on ability to manage an opioid overdose (*n* = 301).

**(a)**			
**Ability Scales**	**Mean (SD) ^b^**		***p*** **-Value ^a^**
	**Current or Past Opioid Use**	**No Current or Past Opioid Use**	
Competence	2.80 (0.89)	2.49 (0.80)	0.003 *
Concerns	2.54 (0.70)	2.70 (0.70)	0.047 *
Readiness	4.20 (0.59)	4.07 (0.64)	0.093
**(b)**			
**Ability Scales**	**Mean (SD) ^c^**		***p*** **-Value ^a^**
	**Exposure**	**No Exposure**	
Competence	2.91 (0.82)	2.40 (0.80)	<0.001 *
Concerns	2.46 (0.71)	2.76 (0.67)	<0.001 *
Readiness	4.28 (0.58)	4.01 (0.63)	<0.001 *

^a^ Mann–Whitney U test (alpha = 0.05). ^b^ In response to the question “*Are you currently using prescription opioid medications or have you been prescribed opioid medications in the past for the treatment of pain or for post-surgery management? Prescription opioid medications may include morphine, hydrocodone, Vicodin^®^, Norco^®^, or others*.” ^c^ In response to the question “*Have you or someone you have known been personally or professionally affected by opioid use disorder (OUD) or substance misuse*?” * Statistically significant at the alpha = 0.05 threshold.

**Table 8:** Predictors of Patient-Pharmacist Naloxone Communication Strategy Preference (n = 301).

	Model 1 ^b^			Model 2 ^c^		
Constructs ^a^	OR	95% CI	*p*-Value	AOR	95% CI	*p*-Value
Awareness of naloxone in general	3.081	1.305–7.275	0.010 *	4.517	1.646–12.397	0.003 *
Awareness of pharmacist provision of naloxone	1.873	0.453–7.747	0.386	2.175	0.472–10.015	0.319
Knowledge about naloxone and opioid overdose	1.013	0.956–1.042	0.344	1.026	0.995–1.058	0.100
General naloxone beliefs	0.859	0.432–1.707	0.665	0.600	0.288–1.251	0.173
Comfort with pharmacist-provided naloxone	2.783	1.257–6.158	0.012 *	3.789	1.584–9.065	0.003 *
Barriers to utilizing pharmacist-provided naloxone	0.898	0.516–1.563	0.703	1.057	0.568–1.969	0.861
Competence in managing opioid overdose	0.479	0.282–0.812	0.006 *	0.353	0.192–0.650	0.001 *
Concerns regarding managing opioid overdose	1.254	0.646–2.434	0.504	1.048	0.515–2.135	0.897
Readiness to manage opioid overdose	1.228	0.580–2.61	0.591	1.638	0.703–3.820	0.253

* Statistically significant at the alph a = 0.05 level. ^a^ Results of logistic regression. OR = Odds Ratio; AOR = Adjusted Odds Ratio; CI = Confidence Interval. Dependent variable = patient-pharmacist naloxone communication strategy preference (targeted offer = 0 (ref), general advertisement or universal offer = 1). Independent variables (constructs) = awareness of naloxone in general (Yes/No), awareness of pharmacist-provided naloxone (Yes/No), mean knowledge score (continuous), general naloxone beliefs mean scale score (continuous), comfort with pharmacist-provided naloxone mean scale score (continuous), barriers to utilizing pharmacist-provided naloxone mean scale score (continuous), competence in managing opioid overdose mean scale score (continuous), concerns regarding managing opioid overdose mean scale score (continuous), readiness to manage opioid overdose mean scale score (continuous). ^b^ Model 1 includes constructs as continuous or categorical predictor variables. Nagelkerke R^2^ = 0.161. ^c^ Model 2 includes constructs as continuous or categorical predictor variables, controlling for respondent characteristics of: age (continuous), gender (dichotomized as male and female), ethnicity (Hispanic/Latino [a] or Not Hispanic/Latino [a]), race (dichotomized as White and All Other Races), education (dichotomized as Less than Bachelor’s Degree and Bachelor’s Degree or Higher), annual household income (dichotomized as Less than $50,000 Annually and $50,000 or more annually), history of opioid use (Yes/No), and life exposure to opioid misuse (Yes/No). Nagelkerke R^2^ = 0.245.

## Data Availability

The data presented in this study are available on request from the corresponding author. The data are not publicly available due to Institutional Review Board restrictions to protect confidentiality of participants.

## Supplementary Materials

The following supporting information can be downloaded at: https://www.mdpi.com/article/10.3390/pharmacy10060171/s1, Supplementary File S1: Survey Instrument.
