# Supplementary material for: Public Perceptions of Community Pharmacy-Based Naloxone Services: A National Cross-Sectional Survey
Source: Pharmacy (Basel). 2022 Dec 9;10(6):171. doi: 10.3390/pharmacy10060171 (PMC9781939; doi:10.3390/pharmacy10060171)
Supplement: Supplementary file 1 [file pharmacy-10-00171-s001.zip › pharmacy-2073194-SI.pdf]

Survey Instrument

Public Perceptions of Community Pharmacy-Based Naloxone Services: A National Cross-Sectional Survey

I. INFORMATION ABOUT YOU

1. Enter your age in years: ___________
2. Please indicate your sex
   ☐Male ☐Female ☐Non-binary ☐Other ☐Prefer not to answer
3. With which race do you identify most closely?
   ☐Black/African American ☐Asian or Pacific Islander
   ☐Native American or Alaska Native ☐White/Caucasian ☐Other
   ☐Prefer not to answer
4. Are you of Hispanic, Latino(a), or Spanish origin?
   ☐Yes ☐No ☐Prefer not to answer
5. What is the highest level of education you have completed?
   ☐Some high school, no diploma
   ☐High school graduate, diploma or the equivalent (for example: GED)
   ☐Some college credit, no degree ☐Trade/technical/vocational training ☐Associate degree
   ☐Bachelor's degree ☐Master's degree ☐Professional degree
   ☐Doctorate degree
6. Please indicate your annual household income:
   ☐Less than $20,000 ☐$20,000-$49,999 ☐$50,000-$99,999 ☐$100,000-$149,999
   ☐$150,000 or more
7. Are you employed in a healthcare profession or non-healthcare profession?
   ☐Healthcare profession ☐Non-healthcare profession ☐Not employed
8. Please indicate the State in which you reside:
   ☐Select from dropdown list of states, territories, and DC ☐Prefer not to answer
9. Please indicate the Zip Code in which you reside: _____________
10. Have you or someone you have known been personally or professionally affected by opioid use disorder (OUD) or substance misuse?
    ☐Yes ☐No
11. Are you currently using prescription opioid medications or have you been prescribed opioid medications in the past for the treatment of pain or for post-surgery management? Prescription opioid medications may include morphine, hydrocodone, Vicodin®, Norco®, or others.
    ☐Yes ☐No

II. GENERAL KNOWLEDGE ABOUT OPIOID OVERDOSE AND NALOXONE

1. Before taking this survey, were you aware of naloxone, also commonly referred to as Narcan®, and what it is used for?
   ☐Yes ☐No
2. What is naloxone used for?
   ☐To reverse the effects of an opioid overdose (e.g. heroin, Oxycodone, Vicodin, methadone)
   ☐To reverse the effects of an amphetamine overdose
   ☐To reverse the effects of a cocaine overdose ☐To reverse the effects of any overdose

3. [Display after Q2 has been answered] Naloxone, or Narcan® is a medication used for the treatment of opioid overdose and can help to reverse an opioid overdose. Were you aware that in all 50 states you can obtain this medication without a prescription by speaking with your pharmacist?
   ☐Yes ☐No
4. Which of the following factors increase the risk of an opioid overdose? Please check all that apply.
   ☐Taking larger than usual doses of opioids
   ☐Increase in opioid purity
   ☐Using an opioid again after not having used for a while
   ☐A long history of opioid use
   ☐Using an opioid when no one else is present around
   ☐Using an opioid again after a detoxification treatment
5. Which of the following are indicators of an opioid overdose? Please check all that apply.
   ☐Having bloodshot eyes ☐Slow or shallow breathing ☐Unresponsive
   ☐Lips, hands or feet turning blue ☐Loss of consciousness ☐Deep snoring
   ☐Very small pupils ☐Agitated behavior ☐Rapid heartbeat
6. Which of the following should be done when managing an opioid overdose? Please check all that apply.
   ☐Stay with the person until an ambulance arrives
   ☐Call an ambulance
   ☐Inject the person with salt solution or milk
   ☐Give mouth to mouth resuscitation
   ☐Give stimulants (e.g. cocaine or black coffee)
   ☐Check for breathing
   ☐Give naloxone (opioid overdose antidote)
   ☐Put the person in a bath of cold water
   ☐Check for blocked airways (nose and mouth)
   ☐Put the person in bed to sleep it off
   ☐Place the person in the recovery position (on their side with mouth clear)
7. How can Naloxone be administered? Please check all that apply.
   ☐Into the nose (intranasal) ☐Into a muscle (intramuscular) ☐Into a vein (intravenous)
   ☐Under the skin (subcutaneous) ☐Swallowing a liquid ☐Swallowing a tablet
   ☐Don't know
8. How long does naloxone take to start having an effect?
   ☐2-5 minutes ☐6-10 minutes ☐11-20 minutes ☐21-40 minutes
   ☐Don't know
9. How long do the effects of naloxone last for?
   ☐Less than 20 minutes ☐About 1 hour ☐1 to 6 hours ☐6 to 12 hours
   ☐Don't know
10. Which statements are CORRECT about naloxone and opioid overdose? Please check all that apply.
    ☐If the first dose of naloxone has no effect a second dose can be given
    ☐There is no need to call for an ambulance if I know how to manage an overdose
    ☐Someone can overdose again even after having received naloxone
    ☐Naloxone can provoke withdrawal symptoms
    ☐After recovering from an opioid overdose, the person must not take any heroin, but it is OK for them to drink alcohol or take sleeping pills

III. GENERAL OPINIONS ABOUT NALOXONE

1. On a scale of 1-5, with 1 being strongly disagree and 5 being strongly agree, please indicate your level of agreement or disagreement with the following statements.

Questions	(1) Strongly Disagree	Disagree	Neutral	Agree	(5) Strongly Agree
Naloxone provides opioid users with a second chance at life	O	O	O	O	O
Naloxone may cause MORE opioid overdoses	O	O	O	O	O
Having naloxone available ENABLES people who misuse opioids	O	O	O	O	O
Naloxone should be made available to anyone concerned about opioid overdose	O	O	O	O	O
If my pharmacist speaks to me about naloxone, I would like to be moved to a private area in the pharmacy	O	O	O	O	O
If my doctor prescribed me opioids, I would NOT need naloxone	O	O	O	O	O

IV. HOW TO RECEIVE NALOXONE FROM THE PHARMACY

1. On a scale of 1-5, with 1 being strongly disagree and 5 being strongly agree, please indicate your level of agreement or disagreement with the following statements.

   What things make it DIFFICULT to receive naloxone from the pharmacy?

Questions	(1) Strongly Disagree	Disagree	Neutral	Agree	(5) Strongly Agree
Cost of naloxone is too high	O	O	O	O	O
It is uncomfortable asking the pharmacist for naloxone	O	O	O	O	O
It is uncomfortable asking the pharmacist for naloxone	O	O	O	O	O
Not wanting my family/friends to know that I was getting naloxone	O	O	O	O	O
Not wanting people I know from my community/neighborhood to see me getting naloxone	O	O	O	O	O
Fear of the pharmacist having a negative view of me if I ask for naloxone	O	O	O	O	O
It will take too long to receive naloxone from the pharmacy, e.g. long wait times	O	O	O	O	O

2. What do you think would be the BEST way for a pharmacist to ask you if you wanted naloxone when you pick up a prescription for opioid pain medications?
   - ☐ Universal offer – pharmacist tells all customers at the pharmacy that naloxone is available
   - ☐ Targeted offer – pharmacist tells only those customers at higher risk of overdose about naloxone
   - ☐ General advertisement – pharmacists posts a flyer so that all customers in the pharmacy are aware naloxone is available

V. OPINIONS ABOUT PHARMACISTS AND NALOXONE

1. On a scale of 1-5, with 1 being strongly disagree and 5 being strongly agree, please indicate your level of agreement or disagreement with the following statements.

**Questions**	**(1) Strongly Disagree**	**Disagree**	**Neutral**	**Agree**	**(5) Strongly Agree**
I am confident that pharmacists have sufficient KNOWLEDGE to dispense naloxone	○	○	○	○	○
I am confident that pharmacists have sufficient TRAINING to dispense naloxone	○	○	○	○	○
I support community pharmacists dispensing naloxone	○	○	○	○	○
I prefer receiving naloxone from my pharmacist rather than my doctor	○	○	○	○	○
I would only get naloxone from my pharmacy if my doctor recommended it first	○	○	○	○	○
If I received naloxone from my pharmacy, I would NOT want them to tell my doctor	○	○	○	○	○
I prefer to receive naloxone from a more clinical setting, like a doctor's office	○	○	○	○	○
The pharmacy is an appropriate place to provide naloxone	○	○	○	○	○
I worry how the pharmacy will handle my naloxone records	○	○	○	○	○
There is not enough privacy in the pharmacy	○	○	○	○	○
I would prefer to receive naloxone from an anonymous source, like the public health department	○	○	○	○	○

VI. YOUR ABILITY TO MANAGE AN OPIOID OVERDOSE

On a scale of 1-5, with 1 being strongly disagree and 5 being strongly agree, please indicate your level of agreement or disagreement with the following statements.

1. Competencies to Manage an Opioid Overdose

Questions	(1) Strongly Disagree	Disagree	Neutral	Agree	(5) Strongly Agree
I already have enough information about how to manage an overdose	O	O	O	O	O
I am already able to inject naloxone into someone who has overdosed	O	O	O	O	O
I would be able to check that someone who has overdosed was breathing properly	O	O	O	O	O
I am going to need more training before I would feel confident to help someone who has overdosed	O	O	O	O	O
I would be able to perform mouth to mouth resuscitation to someone who has overdosed	O	O	O	O	O
I would be able to perform chest compressions to someone who has overdosed	O	O	O	O	O
If someone overdoses, I would know what to do to help them	O	O	O	O	O
I would be able to place someone who has overdosed in the recovery position	O	O	O	O	O
I know very little about how to help someone who has overdosed	O	O	O	O	O
I would be able to deal effectively with an overdose	O	O	O	O	O

2. Concerns About Managing an Opioid Overdose

Questions	(1) Strongly Disagree	Disagree	Neutral	Agree	(5) Strongly Agree
I would be afraid of giving naloxone in case the person becomes aggressive afterwards	O	O	O	O	O
I would be afraid of doing something wrong in an overdose situation	O	O	O	O	O

I would be reluctant to use naloxone for fear of causing withdrawal symptoms	○	○	○	○	○
I would be concerned about calling emergency services in case the police come around	○	○	○	○	○
If I tried to help someone who has overdosed, I might accidentally hurt them	○	○	○	○	○
I would feel safer if I knew that naloxone was around	○	○	○	○	○
I would be afraid of suffering a needle stick injury if I had to give someone a naloxone injection	○	○	○	○	○
Needles frighten me and I wouldn't be able to give someone an injection of naloxone	○	○	○	○	○

3. Readiness to Intervene in an Opioid Overdose

**Questions**	**(1) Strongly Disagree**	**Disagree**	**Neutral**	**Agree**	**(5) Strongly Agree**
Everyone at risk of witnessing an overdose should be given a naloxone supply	○	○	○	○	○
I couldn't just watch someone overdose, I would have to do something to help	○	○	○	○	○
If someone overdoses, I would call an ambulance but I wouldn't be willing to do anything else	○	○	○	○	○
Family and friends of drug users should be prepared to deal with an overdose	○	○	○	○	○
If I saw an overdose, I would panic and not be able to help	○	○	○	○	○
If I witnessed an overdose, I would call an ambulance straight away	○	○	○	○	○
I would stay with the overdose victim until help arrives	○	○	○	○	○
If I saw an overdose, I would feel nervous, but I would still take the necessary actions	○	○	○	○	○
I will do whatever is necessary to save someone's life in an overdose situation	○	○	○	○	○

If someone overdoses, I want to be able to help them	○	○	○	○	○

VII. CONCLUSION

Thank you so much for your time! We appreciate your thoughts and feedback regarding naloxone. If you would like more information regarding Narcan, feel free to visit https://www.narcan.com/
